# Financing Long-term Care: Lessons From Japan

**DOI:** 10.15171/ijhpm.2019.35

**Published:** 2019-05-29

**Authors:** Naoki Ikegami

**Affiliations:** St. Luke’s International University, Tokyo, Japan.

**Keywords:** Long-term Care, Social Insurance, Eligibility Level, Benefit Package, Entitlements

## Abstract

Long-term care (LTC) must be carefully delineated when expenditures are compared across countries because how LTC services are defined and delivered differ in each country. LTC’s objectives are to compensate for functional decline and mitigate the care burden of the family. Governments have tended to focus on the poor but Germany opted to make LTC universally available in 1995/1996. The applicant’s level of dependence is assessed by the medical team of the social insurance plan. Japan basically followed this model but, unlike Germany where those eligible may opt for cash benefits, they are limited to services. Benefits are set more generously in Japan because, prior to its implementation in 2000, health insurance had covered long-stays in hospitals and there had been major expansions of social services. These service levels had to be maintained and be made universally available for all those meeting the eligibility criteria. As a result, efforts to contain costs after the implementation of the LTC Insurance have had only marginal effects. This indicates it would be more efficient and equitable to introduce public LTC Insurance at an early stage before benefits have expanded as a result of ad hoc policy decisions.

Long-term care (LTC) has been defined by the Institute of Medicine as “a variety of ongoing health and social services provided for individuals who need assistance on a continuing basis because of physical or mental disability. Service can be provided in an institution, the home, or community, and include informal services provided by professionals or agencies.”^1^ Disability may occur at any age so LTC is not restricted to the elderly. However, disability generally increases with age, so the need for LTC will increase as the population ages.^2^

This Editorial will first explain why we must carefully define LTC before comparing expenditures across countries.^3^ Next, it will analyze LTC goals and government responsibility in the light of Germany’s LTC social insurance of 1995/1996. Next, it will describe the policy framework within which Japan introduced its public LTC insurance in 2000, and the subsequent efforts made to contain costs. It concludes with possible lessons for policy-makers of other nations who contemplate establishing a public LTC insurance.

## Basic Issues in Long-term Care

### Delineating Long-term Care Expenditures

LTC consists mainly of assistance in activities of daily living, including both “body-touch” care such as for bathing and toileting, and “non-body-touch” care such as for meal preparation and cleaning. How LTC services are defined and delivered differ in each country. For example, in the United States, “nursing homes” provide post-acute care, including rehabilitation therapy, along with custodial care, but in Japan, it is restricted to the latter and the residents expect to remain until they die. Similarly, visiting nurse services are focused on post-hospital discharge care in the United States, whereas in Japan, routine check-up visits to frail elders compose a major part of their activities.

From the above, it is clear that international comparisons of LTC expenditures cannot be made based on the data listed as such in the government statistics. This is why we made the following decisions when we compared LTC expenditures in Australia, England, Germany, Italy, Japan, Sweden, and the United States.^4^ First, we focused only on public expenditures. Private expenditures would be impossible to estimate because LTC is closely entwined with daily life. For example, at what point does the domestic servant become a caregiver? Or, the up-scale housing for elders becomes a nursing facility? Second, we focused only on the expenditures of those 65 and over because there are more variations in coverage and spending across countries for those who are younger (for example, in the extent of occupational training). Third, we included items such as care allowances because they are equivalent to cash benefits in LTC insurance.^5^

The results showed that public per capita LTC expenditures for the population 65 and over were, as we had expected, highest in Sweden at $6399 and lowest in the US at $1525 in 2012. However, unexpectedly, the amount for Germany, which has public LTC insurance, was $1803, and less than that of Italy ($1849) and England ($2280), which do not have formal LTC programmes. The difference lay in the composition of LTC expenditure. In Italy and England, cash benefits were over half of the total, while in Germany, it was only a quarter (benefit-in-kind public assistance for nursing home care composed half of the total). This suggests that the key issue in LTC is not necessarily costs, but resource allocation.

### Long-term Care Goals

The first goal of LTC is to compensate for the decline in functional capacity. LTC provides support in the form of in-kind services by care-workers, or cash benefits to purchase services from informal workers, or to give to a family member or a friend who provides the care. In contrast to healthcare, which is mostly delivered by health professionals, as noted above, LTC is predominantly provided by family, neighbours and friends, even in countries with extensive public provision such as Sweden.^6^ Then why is public financing needed? One reason is that the demand for LTC is increasing with the aging of society, while the availability of informal care has decreased as the size of nuclear families and of women employed outside of their homes has increased.^7^ Thus, in order to maintain and support the informal care that is being provided, the care burden of the family care providers must be mitigated. This is the second goal of LTC.

These two goals are unique to LTC and why LTC should be made independent from the health and social service sectors. The assessment of need must not be left to physicians because the LTC’s objective lies in providing support for activities of daily living, and not in treating the disease or injury. Nor would it seem fair or efficient to wait until all assets have been exhausted before services are made available. The provision of LTC services could lead to earlier and better planned hospital discharges, and to less inappropriate admissions by facilitating coordination, and in difficult cases, by integrating with other sectors.^8^

When making LTC services available, policy-makers must decide the extent to which LTC should include services for preventing functional decline or improving performance. These services could take the form of promoting exercise and social activities in the community, and extend to maintenance rehabilitation for mitigating functional decline. This might strengthen public support because more people would benefit from the programme. However, it could lead to reducing the services available for heavy care users with no assurance that such preventive activities would reduce future LTC costs.^9^

### Government’s Responsibility in Long-term Care

The government’s responsibility in LTC was historically limited to providing institutional care for the poor who do not have any family. This minimalist approach is no longer taken in high-income countries and care has shifted to the community. However, in countries that finance LTC by taxes, with the exception of the Nordic countries, services still tend to be focused on the poor. The extent to which an applicant is entitled to services is left to the discretion of the social worker in charge. Even if social services are made a universal entitlement, geographical disparity is likely to persist.^10^ Local governments typically contract LTC services with provider organizations that submit the best proposal. This contracting can lead to discontinuity in service provision when contracts are given to another provider. Services are allocated based on the relative need of the applicant, who is often not able to choose the provider. Community residents who newly apply for LTC services may be placed on a waiting list, even when they have more needs than the current service recipients.

Germany sought to resolve these issues by moving from a tax-based to a social-insurance-based LTC in 1995/1996.^11^ Eligibility is assessed by nationally uniform standards based on the amount of care time the applicant needs. Benefits are set for five levels. Those eligible can choose between receiving the benefits in cash or services. There is no coinsurance (partial payment) or copayment (a set amount) if services are chosen. However, the benefits do not cover bed and board costs in nursing homes so they must be paid out-of-pocket. If this is not possible, then it will be partially or fully paid by public assistance. The contribution rate of the LTC insurance was initially set at 1.7% of gross income, and has increased to 2.55% in 2017, with an additional 0.25% paid by those 23 and over who do not have a child.^12^ This increase has mitigated erosions of benefits from price hikes. The German LTC insurance is the same as its social health insurance in that everyone is entitled to the same level of benefits, regardless of income or assets, and also in having a free choice of provider. However, it differs in that, first, the amount of benefits is determined by the eligibility level; second, the level is evaluated by the medical team of the insurance plan, and not by the patient’s attending physician; and third, it is not the physician, but the recipient who chooses the services. The first two are similar to the process in social services, but the third is similar to a market exchange.

The following issues still remain. The first is in equating the burden of caring for those with behavioral problems who require 24/7 supervision, with those who require only physical assistance. The second is the quality of services. Users would be able to evaluate the friendliness of the staff and the facility’s amenity level, but not whether the functional status of its residents has declined less, or whether the number of emergency hospital visits was lower, than the national or regional average. This issue can be resolved by mandating the use of inter*RAI* instruments for drawing care plans. Based on the assessment data collected, the LTC facility’s Quality Indicators can be calculated and the results made publicly available.^13,14^

## Japan’s Long-term Care Insurance

### History and Basic Design

Under the old civil code of Japan, the eldest son inherited the family assets, while his wife as the daughter-in-law had the legal and moral obligation to care for her in-law parents. The new civil code enacted in 1947 annulled this obligation and gave equal rights and responsibilities to all children. Still, it continued as a social norm until recent times. The public LTC insurance does not explicitly state that mitigating the care burden of the family, and in particular, that of the daughter-in-law, is its objective. However, this was the underlying social policy issue.^15^

The design and scope of the LTC insurance owes much to policy decisions made in the past. The road towards LTC insurance began in 1973 when “free healthcare” (without coinsurance) was introduced for those 70 and over (and for persons over 65 with disability). Free healthcare had been initiated by populist prefectural governors in the late 1960s. Before then, although everyone was covered by health insurance, most elders had to pay a 50% coinsurance. After the implementation of free healthcare, the use of healthcare by elderly patients increased dramatically, and some hospitals began to function as nursing homes. These hospitals relied heavily on revenues from prescribing drugs and ordering laboratory tests. The government responded by introducing a new type of intermediate health facility in 1986, and a new inclusive payment method for LTC hospitals in 1990. However, the latter did not alter the basic fact that maintaining the staffing of physicians and nurses at the hospital level would be a costly method of delivering LTC services.

In the social welfare sector, LTC services had expanded rapidly after the government launched its “Gold Plan” in 1989 in an effort to win back votes for the ruling party after the election losses it had suffered following the introduction of the unpopular consumer tax (VAT).^16^ The Gold Plan led to sharp increases in LTC financing. It turned out to be very popular, so much so that it was extended from a five-year plan to a ten-year plan in 1994. It included a planned increase of full-time equivalent home-helpers from 38 945 to 170 000 between 1989 and 1999; likewise, an increase in the number of adult day care centers from 1615 to 17 000. These targets were generally met.^17^ However, access to services was controlled by the local government’s social welfare offices. This made the process bureaucratic and subject to ad hoc decisions of the official in charge. Priority was given to the poor and to those without family. Most were exempted from paying because user charges were levied based on a sliding scale. There were considerable geographical variations in services because the decision on the extent to which services were to be developed was left to the municipal mayor.

The government decided that a new public LTC insurance would be the best way to resolve these issues. After its legislation passed, the overriding concern of the Ministry of Health and Welfare (MHW, from 2001, Ministry of Health, Labour and Welfare) was the smooth transfer of services to LTC insurance; from the health sector, LTC hospitals, the new type of LTC health facility, and visiting nurse services; from the social service sector nursing homes, day care centres, home-helper services and so forth were respectively transferred. Eligibility levels were composed of five levels of “need support,” and one (later two) lighter level(s) of “need care.” Benefits ranged from 49 700 yen to 358 300 yen per month (or about 450 to 3000 US dollars at current rates) in home and community-based care based on the eligibility level. Users had to pay a 10% coinsurance (capped for those with low-income), and for the cost of food in institutional settings. For service providers, fees were set by the LTC insurance fee schedule which generally reflected the amount and the conditions of payment that had existed before the transfer. In institutional care, the per diem amount varied according to the type of facility and the resident’s eligibility level.

### Efforts to Contain Costs

In 1997, the MHW estimated that LTC insurance expenditures would double from about 4 trillion yen in 2000 to 10.5 trillion yen in 2010, as more services would become available and assuming an annual inflation rate of 3%.^18^ This projected increase was based on the time required to expand services to the level of the Nordic countries. Actual expenditures did not reach 10 trillion yen until 2018, but since there was little inflation, and implementation was delayed for 3 years, these estimates were more or less accurate. However, the financial burden increased because the increase in the gross domestic product was marginal. The contribution rate for those 40 to 64 enrolled in the largest social insurance plan doubled to 1.73% in 2019.^19^ The amount allocated from taxes also doubled as half of LTC insurance expenditures are financed by taxes. In 2012, the per capita LTC expenditures for the population 65 and over in Japan already amounted to $2832, less than half that of Sweden, but still the second highest among the six countries studied.^4^

Because of the fiscal and budgetary pressure, the Ministry of Health, Labour and Welfare implemented several measures to contain costs. First, it decreased provider fees in the 2003 and the 2006 fee schedule revisions. However, their impact on expenditures was marginal because of rapid increase in volume. Aging within the elderly population has had a much greater effect on LTC costs than on healthcare: per capita health expenditures for individuals 90 and over are only 2.4 times of those between 65 and 69, but LTC insurance expenditures are 44 times more.^20-22^ Moreover, LTC service fees are more difficult to contain than healthcare service fees because LTC workers are more likely to leave the LTC sector if their employer were to lower their wages. This was why the government has not been able to decrease fees in the subsequent revisions of the LTC insurance fee schedule.

Second, benefits have been reduced. Bed and board charges for institutional care residents were introduced in 2005. However, these charges have been waived for the elderly with low-incomes (about 40% of the total).^23^ Next, assets were also taken into account in addition to income in 2015: residents with more than 10 million Yen (US$90 000) in a bank account or equivalent amount in liquid assets would be no longer eligible for the waiver on bed and board charges.^24^

Third, the eligibility criteria for the first (lightest) “need care” level were made stricter in 2006. Most of those in the lightest level in the “need care” were transferred to a newly created level in the “need support” category in 2006 with less benefits. Furthermore, in 2016, social services for those in “need support” came to be set by the municipal authority, and not by the beneficiaries themselves. However, cost savings from these measures are likely to be marginal. Although the number of beneficiaries in the “need support” levels composes a quarter of the total, their expenditures amounted to only 6%.^25^ The eligibility criteria and the benefit amounts for the rest of the beneficiaries have remained the same

Lastly, the government increased the coinsurance rate from 10% for all, to 20% in 2015, and to 30% in 2018 for those in the highest income and/or asset level. However, fewer than 10% of the beneficiaries are estimated to be paying the 30% coinsurance because the income levels of the elders are generally low.^26^ Thus, the extent to which expenditures have been transferred to the service users is marginal.

### Should LTC Insurance Be Introduced?

Japan’s experience of increasing LTC insurance expenditures, and of the limited effect of cost containment measures, may convince policy-makers not to introduce similar entitlement programs. However, they should note the following. First, even if LTC insurance had not been implemented, costs would still have increased because of the ad hoc policy decisions made in the past and because of the aging of the population. Second, resources are now more equitably allocated based on objective eligibility criteria. Third, LTC insurance contributions have become a new revenue source to which people appear to be more willing to contribute than paying higher consumer taxes.

In retrospect, it would have been fiscally more responsible had benefits not been set so generously when LTC insurance was implemented. However, services had been expanded as a result of the policy decisions made by politicians who found that they were popular with the public. These services became an entitlement, which meant that the MHW had to continue their delivery at the same level as before the implementation of the LTC insurance. Although the generous levels of benefits may be unique to Japan, other countries that have been funding LTC through mechanisms such as care allowance and/or have left decisions to local governments would be in a similar situation. This implies that the sooner a public LTC insurance is introduced, the better it would be from both the equity and the fiscal perspectives. Middle-income countries which have achieved universal health coverage should contemplate establishing a LTC system before ad hoc provisions to win popular support become firmly entrenched.^27^

## Ethical issues

Not applicable.

## Competing interests

Author declares that he has no competing interests.

## Author’s contribution

NI is the single author of the paper.
